# Antibiotic Prescribing to Patients with Infectious and Non-Infectious Indications Admitted to Obstetrics and Gynaecology Departments in Two Tertiary Care Hospitals in Central India

**DOI:** 10.3390/antibiotics9080464

**Published:** 2020-07-30

**Authors:** Anna Machowska, Kristoffer Landstedt, Cecilia Stålsby Lundborg, Megha Sharma

**Affiliations:** 1 Department of Global Public Health, Health Systems and Policy, Karolinska Institutet, Tomtebodavägen 18A, 17177 Stockholm, Sweden; anna.machowska@ki.se (A.M.); landstedt_n@hotmail.com (K.L.); Cecilia.Stalsby.Lundborg@ki.se (C.S.L.); 2 Department of Pharmacology, Ruxmaniben Deepchand Gardi Medical College, Ujjain 456006, India

**Keywords:** antibiotics, obstetrics and gynaecology, inpatients, surgical prophylaxis, bacterial infections, fixed dose combinations of antibiotics, Central India

## Abstract

*Background*: Patients admitted to obstetrics and gynaecology (OBGY) departments are at high risk of infections and subsequent antibiotic prescribing, which may contribute to antibiotic resistance (ABR). Although antibiotic surveillance is one of the cornerstones to combat ABR, it is rarely performed in low- and middle-income countries. *Aim*: To describe and compare antibiotic prescription patterns among the inpatients in OBGY departments of two tertiary care hospitals, one teaching (TH) and one nonteaching (NTH), in Central India. *Methods*: Data on patients’ demographics, diagnoses and prescribed antibiotics were collected prospectively for three years. Patients were divided into two categories- infectious and non-infectious diagnosis and were further divided into three groups: surgical, nonsurgical and possible-surgical indications. The data was coded based on the Anatomical Therapeutic Chemical classification system, and the International Classification of Disease system version-10 and Defined Daily Doses (DDDs) were calculated per 1000 patients. *Results*: In total, 5558 patients were included in the study, of those, 81% in the TH and 85% in the NTH received antibiotics (*p* < 0.001). Antibiotics were prescribed frequently to the inpatients in the nonsurgical group without any documented bacterial infection (TH-71%; NTH-75%). Prescribing of broad-spectrum, fixed-dose combinations (FDCs) of antibiotics was more common in both categories in the NTH than in the TH. Overall, higher DDD/1000 patients were prescribed in the TH in both categories. *Conclusions*: Antibiotics were frequently prescribed to the patients with no documented infectious indications. Misprescribing of the broad-spectrum FDCs of antibiotics and unindicated prescribing of antibiotics point towards threat of ABR and needs urgent action. Antibiotics prescribed to the inpatients having nonbacterial infection indications is another point of concern that requires action. Investigation of underlying reasons for prescribing antibiotics for unindicated diagnoses and the development and implementation of antibiotic stewardship programs are recommended measures to improve antibiotic prescribing practice.

## 1. Introduction

Antibiotics are life-saving medicines; however, any use of antibiotics, whether indicated or not, contributes to the development and spread of antibiotic resistance (ABR), one of the most pressing global health threats [1,2,3,4,5,6,7]. The consequences of emergence of ABR are severe and include treatment failure of common bacterial infections leading to increased morbidity, mortality and healthcare costs [4,8,9].

Antibiotics are broadly used in healthcare facilities, and are essential in high infection risk departments, where minor and major surgical procedures take place for both the treatment of infections and as perioperative prophylaxis to prevent healthcare-associated infections (HAIs). For example, in obstetrics and gynaecology (OBGY) departments, antibiotics are used to treat common and severe infections prior and during delivery to prevent maternal and neonatal complications, or as prophylaxis before any surgical procedure, such as caesarean section or uterine prolapse operations, but also to treat infections arising from wounds after surgical procedures [10,11].

Antibiotic use can be effectively monitored via prescription surveillance studies. Data from such studies supplemented by information about local resistance patterns can feed into the development of local antibiotic prescribing guidelines. Availability of local guidelines is crucial to prescribe antibiotics appropriately for specific indication and is the cornerstones to improve the use of antibiotics and to slow down the development of ABR [1,12,13]. Despite this, surveillance at healthcare facilities is underperformed, especially in densely populated, low- and middle-income countries (LMICs) like India [10,14]. At the same time, the majority of the preventable maternal deaths occur in LMICs [15], and there is a paucity of studies that assess antibiotic prescribing patterns among patients admitted to the OBGY departments in India. The published studies have not yet presented the antibiotic prescriptions for specific indications [16,17,18].

Prescriptions are reliable and quantifiable source of information for surveillance studies. However, in resource-constrained healthcare settings, patient information, including prescriptions, are generally not computerised and are often documented manually using paper records only. The lack of automated systems for patients’ data entry makes prescription surveillance an expensive and cumbersome process and is one of the contributors to the delay in the development of contextualised antibiotic prescribing guidelines.

In India, health services are provided in both public- and private-sector healthcare facilities. The private sector provide healthcare to more than 65% households, and the major part of antibiotic use in the country can be attributed to private sector hospitals [19,20,21]. Despite this, limited studies have been conducted on antibiotic prescribing at the Indian private healthcare sector, and the few conducted showed overall high antibiotic prescribing [2,16,17]. These findings are in line with the available evidence of the high use of antibiotics and presence of various multidrug-resistant bacterial strains in the country [18,22]. Therefore, in the present study, we describe and compare antibiotic prescription patterns among the patients admitted to OBGY departments of two, one teaching (TH) and one nonteaching (NTH), tertiary care hospitals in Central India.

## 2. Results

**Figure 1:**
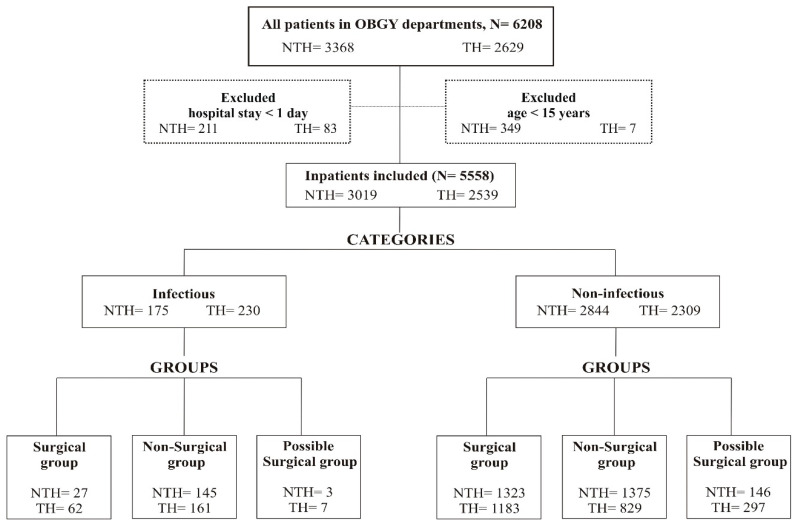
Patients’ categorisation based on the recorded indications in the Obstetrics and Gynaecology departments of two private-sector hospitals, the TH and the NTH in Central India Abbreviations: NTH = Nonteaching hospital; OBGY = Obstetrics and Gynaecology departments; TH = Teaching hospital.

A total of 6208 patients were admitted to both hospitals during the study period and 5558 fulfilled the inclusion criteria, that is, 2539 (46%) patients in the TH and 3019 (54%) in the NTH (Figure 1). A high proportion of patients was prescribed antibiotics in both hospitals. This proportion was higher in the NTH than in the TH (85% vs 81% respectively, *p* < 0.001). Both the duration of hospital stays and the duration of antibiotic treatment were significantly longer in the TH (Table 1).

Full-term normal vaginal delivery (FTND, ICD10- O 80.9, nonsurgical group) was the most common indication for admissions in both hospitals, 209/2539 (8%) in the TH and 572/3019 (19%) in the NTH. The next common indications in the TH were uterine prolapse (192, 8%) and vaginal hysterectomy (VH, 182, 7%, surgical group). In the NTH, surgical procedures such as lower segment caesarean section (LSCS, 480, 16%) and abdominal hysterectomy (AH, 328, 11%) were the next most common indications.

Among 192 patients admitted due to uterine prolapse in the TH, more than 70% received antibiotic prescriptions, of which eight patients had a record of confirmed or suspected infectious indication. Out of all patients that underwent LSCS and hysterectomy in the NTH, 11 patients had a record of confirmed and/or suspected infection. More than 95% of the patients that underwent abdominal or VH or LSCS received antibiotics in both hospitals; however, average number of days of antibiotic treatment was longer in the TH than in the NTH.

Although no significant difference was observed in the indications among the patients admitted to the TH and NTH, it seems that more ill patients were admitted to the TH. This may be due to the wider rural catchment area of the TH, leading to more patients from villages being admitted to the hospital. Study shows that such patients often sought healthcare late [23].

### 2.1. The Infectious and Non-Infectious Categories

The 5558 patients included in the analysis were divided into infectious (TH-230, NTH-175) and non-infectious (TH-2309, NTH-2844) diagnosis categories and were further divided into surgical, nonsurgical, and possible surgical groups (Figure 1, Table 2). The total number of prescriptions in both infectious and non-infectious diagnosis categories was higher in the TH across all three diagnosis groups. The length of hospital stay and duration of antibiotic treatment for the patients in the non-infectious diagnosis category were significantly longer in the TH than in the NTH, regardless of whether surgery was performed or not. The number of patients who received antibiotics for one day was less than 5% at both hospitals, in surgery groups of both infectious and non-infectious diagnosis categories. The length of antibiotic administration for one day can be a proxy for antibiotic prophylaxis, which is indicated for the surgical procedures (Table 2).

Seventy-six per cent of patients in the infectious diagnosis category were in the nonsurgical group (TH-161, NTH-145), and 88% (269/306) of these were prescribed antibiotics during their hospital stay. In the non-infectious diagnosis category, 43% of patients (2204/5153) were classified in the nonsurgical group, (TH-829, NTH-1375) and more than 70% received antibiotics during their hospital stay. The majority (98%) of the inpatients in the possible surgery group had non-infectious indications, but 77% of these patients in the TH and 50% in the NTH were prescribed antibiotics.

### 2.2. Antibiotic Prescribing Patterns in the Categories and Groups

In both the infectious and non-infectious diagnosis categories, across all three groups (surgical, nonsurgical and possible surgical), the use of generic names was more common, and adherence to the National List of Essential Medicines of India (NLEMI) was higher in the TH than in the NTH (Table 3).

In the infectious diagnosis category, nitroimidazole derivatives (23% and 24%) and tetracyclines (19% and 16%), respectively, were the most commonly prescribed in surgical and nonsurgical groups in the TH (Table 3). Overall, in this category, other beta-lactams and FDCs were prescribed to a greater extent in the NTH than in the TH (*p* < 0.001). In the surgical group of the infectious diagnosis category, the FDCs of third-generation cephalosporins accounted for 53% of all prescriptions in the NTH, while FDCs were not prescribed at all in the TH.

In the non-infectious diagnosis category, third-generation cephalosporins and the FDCs accounted for 58% of all prescriptions in the surgical group in the NTH, whereas aminoglycosides (18%), imidazole (17%), and nitroimidazole derivatives (17%) were the most commonly prescribed in the TH. In the nonsurgical group in the TH, extended-spectrum penicillin and imidazole derivatives constituted 52% of all prescriptions, whereas in the NTH, third-generation cephalosporins and FDCs constituted 57% (Table 3).

### 2.3. Antibiotic Prescribing in DDD/1000 Patients

Defined Daily Doses (DDD) per 1000 patients was calculated at the substance level based on the Anatomical Therapeutic Chemical (ATC) methodology. The antibiotics accounting for 90% of all prescriptions (DU90%) for each diagnoses group are presented in Table 4. Overall, higher DDD/1000 patients were prescribed in the TH than in the NTH in both categories. In the TH, regardless of the presence of an infectious indication, doxycycline (in surgical and possible surgical groups) and ampicillin (in the nonsurgical group) were the highest prescribed DDD/1000 patients. In the infectious diagnosis category of the NTH, cefixime was most commonly prescribed in the surgical group, ceftriaxone in the nonsurgical group, and metronidazole in the possible surgical group, whereas in the non-infectious diagnosis category, cefixime was the most frequently prescribed (Table 4).

## 3. Discussion

To the best of our knowledge, this is the first study that describes and compares antibiotic prescription in relation to the presence of infection and surgery indication among the patients admitted to the OBGY departments of two private hospitals in India. Frequent prescribing of antibiotics was common in both hospitals, including overprescribing of the FDCs. Prescribing of antibiotics for unindicated conditions, specifically to the inpatients having non-infectious (bacterial) indications, was observed.

So far, a few studies conducted either at the hospital level or for a shorter period that present antibiotic prescribing in OBGY patients have been published [2,16,17].

The results of our study indicate several trigger factors for the development of ABR. Firstly, an overall high antibiotic prescribing rate, more than 80%, of patients received antibiotics during their hospital stay. This is comparable to the results showed in a study conducted by Sharma et al. (86%), which is also part of the current project [17], but is lower than in a study conducted by Alvarez et al. in a rural hospital in Andhra Pradesh, India (92%) [2]. In both studies, the analysis was conducted for all admitted patients, but indications for prescribed antibiotics were not considered. In the present study, we analysed data corresponding to the indications at both hospitals and observed extensive antibiotic prescribing to the patients without any record of an infectious indication. Secondly, we observed that the patients who underwent surgery were prescribed antibiotics to a higher extent (>85%) than those in the nonsurgical group (>70%). However, prescribing antibiotics for the patients who neither underwent surgery, nor had any signs of infection cannot be explained at present and needs further detailed investigation.

### 3.1. Adherence to Guidelines

The prescriptions in the TH adhered more to the NLEMI than those in the NTH and were more frequently made using generic names. A similar pattern was observed in the infectious diagnosis category in both hospitals. One of the possible explanations for the observed patterns, i.e., higher trade name prescribing in the NTH, might be that the consultants associated with the NTH can run their private clinics, where they are free to meet medical representatives of pharmaceutical companies. These meetings may influence the content of the consultant’s prescriptions. Studies show that medical representatives lure consultants into prescribing antibiotics by the trade name to favour the sale of the products of a particular pharmaceutical company [24]. On the other hand, higher generic name prescribing in the TH can be explained by the hospital policies which restrict the consultants to run private clinics, and to interact with medical representatives. Moreover, the hospital management of the TH prefers to purchase and supply generic medicines to the hospital pharmacy. In addition, a positive impact of the routine academic activity, e.g., continuing medical education regularly held in the TH, might also have motivated consultants towards adhering to the recommendations. These hospital policies could be modified contextually and implemented at other similar settings to rationalise antibiotic prescriptions.

### 3.2. Hospitalisation, Antibiotic Treatment Duration and Costs

Patients admitted to the TH had significantly longer hospital stays and durations of antibiotic treatment, compared to the patients in the NTH (Table 1) and both are risk factors for HAIs. Suspicion or actual presence of HAIs results in antibiotic prescriptions [25]. Data on the prevalence of HAIs was not available for the entire study duration in the OBGY departments of the study hospitals due to the absence of computerised records and limited utilisation of diagnostic facilities. Therefore, it would be inappropriate to comment on the relationship between the HAIs and antibiotic prescribing in the settings. However, the HAI prevalence could be expected to be similar to those in other comparable settings [26].

In-hospital days are directly related to increased number of prescriptions and treatment costs. The differences in the hospitalisation lengths between the hospitals can be explained based on the system of providing services. In the NTH, patients are charged for the hospital stay, whereas all services in the TH the are free of charge. Therefore, patients might have opted to get an early discharge from the NTH mainly due to out-of-pocket expenses to reduce the economic burden [18].

### 3.3. Antibiotic Prescribing in Infectious and Non-Infectious Categories, and Groups

Choice of antibiotics, both at the category and group levels, varied significantly between the hospitals. The FDCs (J01RA*) and third-generation cephalosporins were frequently prescribed in the NTH, constituting more than half of the prescriptions. These FDCs are neither included in the WHO List of Essential Medicines nor in the NLEMI [17]. Combining antibiotic substances to prepare a FDC is often considered as irrational and is a costlier option compared to single medicine [9,17,27]. The prescriptions of broad-spectrum antibiotics and FDCs are examples of misuse and overuse of antibiotics and increase the risk of spread and development of ABR. These issues need an immediate attention of prescribers and policymakers [17,18].

The presence of bacterial infection is an appropriate indication for antibiotic treatment in postoperative procedures, whereas a surgery per se is an indication to receive a single prophylactic dose of antibiotic before or during a routine surgery [28]. The most recent guidelines from the Centre for Disease Control and Prevention state that additional prophylactic antibiotics should not be administered after the surgical incision is closed in clean and clean-contaminated procedures [29].

In our study, contrary to these recommendations, antibiotics were prescribed in the absence of any clinical decision or laboratory confirmation of the presence of an infection. More than 70% of patients in the non-infectious diagnosis category and nonsurgical group were also prescribed antibiotics in both hospitals (Table 2). This number is comparable with a study in an Indian hospital, where nearly 70% of women with severe pre-eclampsia or eclampsia, which is not an indication for antibiotic use, were prescribed antibiotics. However, most of these women underwent emergency LSCS, which can justify the high use of antibiotics [30]. On the other hand, in our study, only 23% of patients had LSCS, which points towards a high percentage of women with unindicated antibiotic use.

Our results from the surgical groups showed that a large proportion of the inpatients who did not have a confirmed infection received antibiotics. Moreover, the duration of prescribing antibiotics exceeded the recommended prophylactic duration among almost all inpatients in the surgery groups of both categories, including patients of elective surgery group (hysterectomy and LSCS) [11,31]. Prescribing antibiotics as treatment is indicated only in cases of postoperative or concomitant infections for procedures such as caesarean sections and hysterectomies [31]. Comparable prescription patterns have previously been presented in two Indian studies where patients were treated with antibiotics for multiple days after caesarean section instead of receiving a single-dose prophylaxis [30,32]. Prescribing antibiotics as a treatment and for extended durations to non-infectious, nonsurgical cases is not recommended and increases not only the risk of the ABR development, but also the treatment cost.

Patients that underwent elective surgeries received antibiotic treatment for multiple days instead of the recommended single dose prophylaxis at both hospitals.

The specific underlying reasons for prescribing antibiotics for extended durations and for unindicated conditions in the present study settings are not clear. However, a survey conducted among 650 surgeons in India also supports our results. The survey reflected that surgeons do prescribe antibiotics for more extended periods than recommended in the standard surgeon’s guidelines and recommendations [33]. A lack of local prescribing guidelines could be one of the underlying reasons for the observed high antibiotic prescribing. Interventions, such as development and successful implementation of relevant antibiotic prophylaxis guidelines and regular prescription audits, would help to reduce antibiotic prescription, as demonstrated for LSCS patients in a single centre study from Serbia [34]. This study showed a significant postintervention decrease in the use of third generation cephalosporins and reduction of treatment cost by 47% [34]. The underlying factors affecting the prescribing patterns at the settings are not known. Therefore, we recommend further investigation in a separate, focused, qualitative study targeting the prescribers at both hospitals.

In the nonsurgical groups at both hospitals, FTND was the most common condition for admission, and the majority of patients who gave birth with FTND also received antibiotic treatment. Antibiotic prescription to FTND patients cannot be explained, as even the prophylactic use of antibiotics in this group is not recommended [35]. A previous study from Ujjain district in India has shown antibiotic prescribing to 87% of FTND patients [36]. An episiotomy is a possible indication for antibiotic prophylaxis in FTND; however, according to a Cochrane review, further studies are required to confirm it [37]. Thus, a targeted study is suggested to investigate the rationality of prescribing antibiotics for FTND.

### 3.4. Strengths and Limitations

The prospective, long term data collection in a situation without a computerised system is the main strength of this study. The data was collected prospectively over three years, which facilitated studying the antibiotic prescribing patterns for an extended period and including the relatively large population of 5558 patients.

Additionally, the data was collected comprehensively for every admitted patient to overcome the selection bias. All diagnoses were checked manually from the patients’ files and patients were divided into the diagnosis groups in consultation with two local obstetricians and gynaecologists for comprehensive categorisation. The medical consultants were not identified at any stage of the study. This method of data collection might have given consultants the freedom to decide on the treatment plan for the patients.

However, this study must be seen in the context of its limitations. First, since the data was collected manually, the possibility of missing data was foreseen. For the missing data, the records in the archive were checked before the analysis. For a small proportion of patients, information on whether the patients were operated on or not could not be retrieved. For such patient records, a third diagnosis group, “possible surgical”, was created to nullify the probable overestimation of antibiotic prescribing. Secondly, the diagnoses (indications) were not validated externally. Finally, the use of personal identification numbers, the presence of inexperienced staff for data collection, high staff turnover and the absence of computerised record systems in hospitals make a comprehensive study like this time-consuming and tedious, which causes a delay in analysis. We are aware that extensive manual checking and adding of the ICD codes and ATC codes for the new FDCs in the data have prolonged the analysis and delayed the presentation. However, the use of human resources is the only way to conduct such detailed studies in resource-constrained settings and leads to a more accurate description of the prescribing patterns. Although the data represented in this paper covers the time from 2008 to 2011, a similar pattern of antibiotic use has been predicted through an extrapolation model by Tamhankar et al. [38] and was observed by Damlin et al. [39]. Thus, it indicates that the patterns presented in our study are similar to those of recent years. This increases the validity of the results of our study in the present context.

## 4. Materials and Methods

### 4.1. Study Setting and Design

This was a prospective, cross-sectional study conducted in the OBGY departments of one TH and one NTH in Central India. The data was collected for all patients admitted to the OBGY departments of both hospitals between March 2008 and April 2011. Detailed information about the study hospitals is presented in other studies that are part of the same larger project [17,18]. In brief, both study hospitals are private-sector tertiary-care hospitals, run by a not-for-profit charitable trust, but differ in mode of providing healthcare services and have different administrative and operative approaches.

The TH is situated in a rural area and provides all medical services free of charge. All medicines are purchased by the hospital management and are dispensed free of cost to all admitted patients. The staff of the TH has a routine to participate in academic activities of continuing medical education in the TH. The NTH is located in a city area of Ujjain where patients are charged for the medical consultancy, hospital stay, and have to purchase the prescribed medicines out of pocket during their hospital stay [18]. Being a part of a charitable trust, the medical services are provided at subsidised rates in the NTH. The salary structure differs between the hospitals, as in the TH, the physicians receive a fixed salary, while the payment for physicians in the NTH is mainly based on the number of patients they admit in the hospital.

### 4.2. Data Collection and Study Population

The data on antibiotic prescribing was collected manually in the OBGY departments of the two study hospitals. A locally developed form was used to collect the data on patients’ demographics, admission and discharge dates, indications or diagnoses, prescribed antibiotics during the hospital stay and at discharge, (name of antibiotic, dose, route of administration, duration and frequency). In addition, brief information about the microbiology testing, date of surgery in case of operated patients, and outcome of the patients (such as discharged, shifted to other ward or referred to other hospital) were also collected. The data was routinely collected by the nurses working in the OBGY wards, the filled forms were cross-checked for completeness and the data was manually entered in the excel file and EpiData software by the trained data entry persons.

The nurses working in the OBGY wards were trained repeatedly by M.S. to collect the data from the patients’ record files in the paper forms. A patient could have more than one diagnosis (indications). All indications, as written in the patients’ files at the time of discharge, were noted in the form. After the patient’s discharge, the filled forms were collected from the wards on daily basis from the TH and on weekly basis from the NTH.

### 4.3. Inclusion Criteria

Female patients who spent at least one night in the OBGY departments and were more than 15 years old were included in the analysis [40,41] (Figure 1).

### 4.4. Data Management and Analysis

The diagnoses were grouped according to the International Statistical Classification of Disease and Related Health Problems-Tenth Revision (ICD-10) [42]. For some patients, the site of the surgery was not specified, or a complaint was recorded as an indication. These unspecified indications or complaints are not classified by ICD-10 code and were thus abbreviated by the authors, e.g., an abdominal hysterectomy was abbreviated as AH. The diagnoses assigned by the consultant and registered in the patients’ file were considered as final and were not validated externally.

Based on the information in patients’ records, the patient data were categorised based on the presence or absence of infectious indications in two categories: infectious diagnosis and non-infectious diagnosis. While categorising, if a patient had both an infectious and a non-infectious diagnosis, then the patient was categorised as infectious. The infectious diagnosis category comprised all patients with confirmed or suspected infectious diseases, including bacterial, viral, and fungal infections, or having any clinical signs of infection such as fever, pus in wound, infected wound. The non-infectious diagnosis category comprised all patients who had no documentation of infectious indication or no clinical signs of any infection, such as anaemia, labour pain, pregnancy, amenorrhoea. In the next step, both the infectious and non-infectious diagnosis categories were further divided into three diagnosis groups: surgical, nonsurgical, and possible surgical (Figure 1). Patients who had an indicated or confirmed surgery status were assigned to the surgical patient group. Patients who had no indication for surgery were included in the nonsurgical group. Patients that had a diagnosis where a surgical procedure was indicated but the status of the surgery was not specified were included in the possible surgical patient group.

Data analysis was performed anonymously using a unique patient code system assigned after completing the data collection. The prescribed antibiotics were categorised as per the ATC classification system according to the WHO Collaborating Centre for Drug Statistics Methodology (WHOCC) [43]. In both hospitals, antibiotics could have been prescribed by either generic or trade names. In case an antibiotic was prescribed by trade name, the corresponding generic name was also entered to facilitate the analysis according to the WHO methodology at the substance level of the ATC. DDD, as suggested by WHOCC, was used as a unit for the analysis [43].

The local antibiotic prescribing guidelines were not available in any of the study hospitals. The prescribing patterns were compared with the NLEMI and the WHO List of Essential Medicines in absence of local or national antibiotic prescribing guidelines for OBGY indications [44,45]. Levels of adherence of prescriptions to the NLEMI were analysed and compared between the hospitals [44]. The NLEMI is relevant for the national context and thus the recommendations from the NLEMI were followed in the present study [44]. For the new FDCs of antibiotics for which ATC codes were not assigned by the WHOCC, the earlier generated codes, J01RA*, were used [17,18].

Data were analysed using Excel, EpiData software, version 3.1 (EpiData Association, Odense, Denmark), STATA software version 15.0 (Stata Corp., College Station, Texas, USA) and SPSS Statistics version 22 (SPSS Inc, Chicago, IL, USA). For continuous variables sum, mean and standard deviations and for categorical variables, frequency and percentage were calculated. Decimals were rounded off to the nearest number. The independent samples *t*-test was used for comparison of continuous variables since the variables followed a normal distribution. For comparisons of categorical values, Pearson chi-square was used. *P* values ≤ 0.001 were considered significant, according to Bonferroni’s correction.

### 4.5. Ethics Approval and Consent to Participate

The ethics committee of Ruxmaniben Deepchand Gardi Medical College, Ujjain, approved the study with the number: 41/2007 and 114/2010. This was an observational study where the data collection was done using patient files, thus no patient consent was needed. The study did not interfere with the patients receiving the treatment, and none of the patients was contacted during the study period. Each patient was given a unique code. The analysis was performed using these unique codes to maintain confidentiality. No exclusions were made by age, sex or other demographic criteria during data collection.

The data are available to all interested researchers upon request made to; The Chairman, Ethics Committee, R. D. Gardi Medical College, Agar Road, Ujjain, Madhya Pradesh, India 456006 (Email: iecrdgmc@yahoo.in, uctharc@bsnl.in), giving all details of the study. The ethical approval numbers 41/ 2007 and 114/2010 are to be quoted along with the request.

## 5. Conclusions

High antibiotic prescribing was observed in OBGY departments in both hospitals; however, it was more common in the NTH than in the TH. Antibiotics were prescribed empirically to the patients without reported clinical infection indications in both hospitals. Patients that underwent elective surgeries received courses of antibiotics for several days, despite the recommendations to prescribe a single-dose preoperative prophylaxis. Broad-spectrum antibiotics, including the new FDCs, were more frequently prescribed and trade names were more commonly used in the NTH than in the TH. This practice can lead to an increase of ABR and needs urgent action.

A multiple-step approach including an antibiotic stewardship program is suggested to address the issue of overprescribing and misprescribing of antibiotics. The feedback of the study results, which were provided to the consultants, is the first step to alter their clinical practice. However, it needs to be combined with the development and implementation of local diagnosis-specific antibiotic prescribing guidelines and recurrent training and educational sessions, which are still lacking at the study site. The antibiotic stewardship needs to be accompanied by longitudinal surveillance of the prescriptions, which requires continuous funding sources, and optimally, computerised patient data collection systems. In addition, qualitative studies among the prescribers need to be performed to explore the underlying reasons for the present prescription patterns in both settings.

## Figures and Tables

**Table 1:** Patient’s characteristics and antibiotic prescribing in the Obstetrics and Gynaecology departments of two private-sector hospitals, teaching (TH) and nonteaching (NTH), in Central India.

Variables	TH	NTH
Total number of patients	2539	3019
Age; mean years (SD)	34 (13) *	30 (11)
Patients prescribed AB; n (%)	2044 (81)	2567 (85) *
Duration of hospital stay; mean days (SD)	8 (9) *	3 (3)
Duration of AB treatment; mean days (SD)	8 (7) *	5 (3)
Total number of prescription days	30,616	12,512

Statistically significant values (*p* < 0.001) are marked with *. Abbreviations: AB = antibiotics; NTH = Nonteaching hospital; OBGY = Obstetrics and Gynaecology departments; SD = standard deviation; TH = Teaching hospital.

**Table 2:** Patients’ demographic and prescription details at the category and group levels among inpatients at the Obstetrics and Gynaecology departments of two private-sector hospitals, the TH and the NTH, in Central India.

**Infectious Diagnosis Category; *n* = 405 (TH = 230, NTH = 175)**						
**Variables**	**Surgical (*n* = 89)**		**Nonsurgical (*n* = 306)**		**Possible Surgical (*n* = 10)**	
	**TH**	**NTH**	**TH**	**NTH**	**TH**	**NTH**
Total patients in each diagnosis group; n	62	27	161	145	7	3
Patients prescribed AB; n (%)	57 (92)	27 (100)	143 (89)	126 (87)	6 (86)	3 (100)
Duration of hospital stay; mean days (SD)	11 (10)	6 (3)	6 (5) *	3 (3)	13 (5)	3 (2)
Duration of AB treatment; mean days (SD)	9 (7)	6 (3)	6 (4) *	3 (2)	9 (3)	4 (2)
AB prescriptions in each diagnosis group; n	974	186	1592	479	91	16
AB prescriptions by generic name; n (%)	182 (19)	17 (9)	393 (25) *	15 (3)	2 (2)	0 (0)
Types of AB substances prescribed; n	17	22	18	34	10	3
AB prescribed using generic name; n (%)	7 (41)	2 (9)	8 (44)	3 (9)	1 (10)	0 (0)
Prescriptions of AB found in the NLEMI; n (%)	681 (70)	112 (60)	1084 (68) *	271 (57)	54 (59)	3 (19)
Number of patients prescribed AB for one day	2 (3)	0 (0)	4 (2)	12 (8)	0 (0)	0 (0)
**Non-infectious diagnosis category; *n* = 5153 (TH = 2309, NTH = 2844)**						
**Variable**	**Surgical (*n* = 2506)**		**Nonsurgical (*n* = 2204)**		**Possible Surgical (*n* = 443)**	
	**TH**	**NTH**	**TH**	**NTH**	**TH**	**NTH**
Total patients in each diagnosis group; n	1183	1323	829	1375	297	146
Patients prescribed AB; n (%)	1030 (87)	1268 (95) *	592 (71)	1025 (75)	216 (73)	119 (82)
Duration of hospital stay; mean days (SD)	13 (10) *	6 (3)	6 (6) *	3 (3)	10 (9) *	4 (4)
Duration of AB treatment; mean days (SD)	9 (8) *	6 (3)	6 (4) *	3 (2)	8 (7) *	4 (3)
AB prescriptions in each diagnosis group; n	19,024	7785	5165	3484	3770	562
AB prescriptions by generic name; n (%)	5757 (30) *	303 (4)	2439 (47) *	89 (3)	1040 (28) *	30 (5)
Types of AB prescribed; n	30	51	22	46	29	32
AB prescribed using generic name; n (%)	12 (43) *	5 (9)	8 (36)	5 (11)	9 (31)	2 (6)
Prescriptions of AB found in the NLEMI; n (%)	14,920 (78)	3881 (50)	4224 (82) *	2010 (57)	2917 (77) *	282 (50)
Number of patients prescribed AB for one day	29 (2)	72 (5)	15 (2)	111 (8)	10 (3)	9 (6)

Statistically significant values (*p* < 0.001) are marked with *. Abbreviations: AB = antibiotics; NLEMI = National List of Essential Medicines India; NTH = Nonteaching hospital; OBGY = Obstetrics and Gynaecology departments; SD = standard deviation, TH = Teaching hospital.

**Table 3:** Classes and subclasses of prescribed antibiotics at the category and group level in the Obstetrics and Gynaecology departments of two private-sector hospitals, the TH and the NTH, in Central India.

**Infectious diagnosis category; *n* = (TH-230, NTH-175)**						
**Antibiotic Class**	**Surgical**		**Nonsurgical**		**Possible Surgical**	
	TH n (%)	NTH n (%)	TH n (%)	NTH n (%)	TH n (%)	NTH n (%)
Total prescriptions	974	186	1592	479	91	16
Tetracyclines; J01A, J01AA	189 (19) *	1 (1)	251 (16) *	3 (1)	21 (23)	-
Beta-lactam AB, penicillin; J01C	83 (9)	20 (11)	237 (15)	69 (14)	7 (8)	3 (19)
Extended-spectrum penicillin; J01CA	66 (7)	-	202 (13) *	13 (3)	7 (8)	-
Comb. of penicillin incl. Beta-lactamase AB; J01CR	17 (2) *	20 (11)	29 (2) *	56 (12)	-	3 (19)
Other Beta-lactam; J01D	100 (10) *	67 (36)	103 (6) *	174 (36)	10 (11)	-
3rd gen. cephalosporins; J01DD	90 (9) *	55 (30)	103 (6) *	161 (34)	10 (11)	-
Aminoglycoside; J01G	104 (11)	17 (9)	180 (11) *	21 (4)	-	-
Other aminoglycosides; J01GB	104 (11)	17 (9)	180 (11) *	18 (4)	-	-
Quinolones; J01M, J01MA	122 (13)	27 (15)	241 (15)	48 (10)	9 (10)	-
Fixed dose combination of AB; J01R, J01RA *	-	43 (23)	-	126 (26)	-	13 (81)
Other AB; J01X	129 (13)	9 (5)	183 (11) *	20 (4)	2 (2)	-
Imidazole derivatives; J01XD	129 (13)	9 (5)	183 (11) *	20 (4)	2 (2)	-
Nitroimidazole derivatives; P01A, P01AB	224 (23) *	2 (1)	390 (24) *	4 (1)	30 (33)	-
**Non-Infectious Diagnosis Category *n* = (TH-2309, NTH-2844)**						
**Antibiotic Class**	**Surgical**		**Nonsurgical**		**Possible Surgical**	
	TH n (%)	NTH n (%)	TH n (%)	NTH n (%)	TH n (%)	NTH n (%)
Total prescriptions	19,024	7785	5165	3484	3770	562
Tetracyclines; J01A, J01AA	1777 (9) *	53 (1)	158 (3) *	25 (1)	350 (9) *	3 (1)
Beta-lactam AB, penicillin; J01C	2791 (15) *	606 (8)	2156 (42) *	543 (15)	400 (11)	48 (9)
Extended-spectrum penicillin; J01CA	2466 (13) *	61 (1)	1858 (36) *	296 (8)	304 (8) *	6 (1)
Comb. of penicillin incl. Beta-lactamase AB; J01CR	279 (1) *	545 (7)	290 (6)	247 (7)	93 (2) *	42 (7)
Other Beta-lactam; J01D	1316 (7) *	2689 (35)	390 (8) *	1410 (40)	478 (13) *	182 (32)
1st gen. cephalosporins; J01DB	137 (1)	57 (1)	23 (0) *	53 (2)	59 (2)	3 (1)
2nd gen. cephalosporins; J01DC	12 (0) *	297 (4)	-	151 (4)	19 (1) *	31 (5)
3rd gen. cephalosporins; J01DD	1167 (6) *	2335 (30)	363 (7) *	1193 (34)	400 (11) *	148 (26)
Sulfonamide with trimethoprim; J01E, J01EE	131 (1) *	8 (0)	20 (0)	10 (0)	46 (1)	-
Macrolides, lincosamides J01F	7 (0) *	34 (0)	12 (0) *	34 (1)	18 (1)	-
Macrolides; J01FA	7 (0) *	23 (0)	5 (0) *	29 (1)	9 (0)	-
Aminoglycoside; J01G	3379 (18) *	528 (7)	734 (14) *	156 (4)	694 (18) *	58 (10)
Other aminoglycosides; J01GB	3379 (18) *	522 (7)	734 (14) *	156 (4)	694 (18) *	58 (10)
Quinolones; J01M, J01MA	3071 (16)	1317 (17)	432 (8) *	403 (11)	544 (14)	64 (11)
Fixed dose combination of AB; J01R, J01RA *	17 (0) *	2217 (28)	-	801 (23)	2 (0) *	159 (28)
Other AB; J01X	3364 (18) *	300 (4)	804 (16) *	100 (3)	645 (17) *	45 (8)
Imidazole derivatives; J01XD	3320 (17) *	300 (4)	804 (16) *	100 (3)	636 (17) *	40 (7)
Drugs for treatment of tuberculosis; J04A, J04AM	-	3 (0)	-	-	-	3 (1)
Nitroimidazole derivatives; P01A, P01AB	3162 (17) *	30 (0)	459 (9) *	10 (0)	593 (16)	-

Statistically significant values (*p* < 0.001) are marked with *. Abbreviations: AB = antibiotics; ATC = The Anatomical Therapeutic Chemical Classification System; *n* = number of prescriptions; NTH = Non-teaching hospital; OBGY = Obstetrics and Gynaecology departments; TH = Teaching hospital.

**Table 4:** DDD/1000 patients for the most prescribed antibiotic substances (DU90%) at category and group level in the Obstetrics and Gynaecology departments of two private-sector hospitals, the TH and the NTH, in Central India.

Name of the Antibiotic Substance		Infectious Diagnosis Category						Non-Infectious Diagnosis Category					
		Surgical Group		Nonsurgical Group		Possible Surgical Group		Surgical Group		Nonsurgical Group		Possible Surgical Group	
		TH	NTH	TH	NTH	TH	NTH	TH	NTH	TH	NTH	TH	NTH
		**Total DDDs/1000 patients**											
		14,587	9693	8668	3532	10,454	6856	13,132	7134	4696	2756	10,243	4540
	**ATC**	**DDDs/1000 patients**											
Doxycycline	J01AA02	5855	-	2988	-	4286	-	2848	-	-	-	2125	-
Ampicillin	J01CA01	972	-	862	-	-	-	1716	-	1734	14	846	-
Amoxicillin	J01CA04	-	-	-	69	1071	-	-	-	314	126	-	-
Amoxicillin + Clavulanic acid	J01CR02	-	1376	-	247	-	2400	-	623	-	222	-	429
Piperacillin + Tazobactam	J01CR05	-	-	-	67	-	-	-	-	-	-	-	33
Ampicillin + Cloxacillin	J01CR50	-	-	-	22	-	-	-	-	180	-	-	-
Cefalexin	J01DB01	-	-	-	-	-	-	-	-	-	7	-	-
Cefuroxime	J01DC02	-	341	-	123	-	-	-	312	-	139	-	224
Cefotaxime	J01DD01	839	148	301	45	-	-	423	187	179	68	513	91
Ceftazidime	J01DD02	-	93	-	34	-	-	-	-	-	-	-	-
Ceftriaxone	J01DD04	-	1259	-	755	1200	-	-	737	-	350	-	479
Cefixime	J01DD08	-	2222	-	448	-	-	-	1548	-	530	-	788
Cotrimoxazole*	J01EE01	-	-	-	-	326	-	-	-	-	-	-	-
Cefpodoxime	J01DD13	-	-	-	-	-	-	-	194	-	216	-	377
Gentamicin	J01GB03	1075	-	654	37	-	-	1844	179	567	58	1395	105
Amikacin	J01GB06	-	481	-	69	-	-	-	226	-	65	290	274
Azithromycin	J01FA10	-	-	-	184	-	-	-	-	-	-	-	-
Ofloxacin	J01MA01	-	-	-	-	-	-	-	-	-	-	-	82
Ciprofloxacin	J01MA02	1261	459	779	444	-	-	2098	287	289	167	1517	140
Norfloxacin	J01MA06	520	-	615	-	1000	-	-	-	191	-	325	-
Levofloxacin	J01MA12	-	1111	-	131	-	-	-	1083	-	174	-	340
Moxifloxacin	J01MA14	-	-	-	-	-	-	-	118	-	-	-	60
Ceftazidime + Tazobactam	J01RA*82	-	-	-	-	-	-	-	-	-	-	-	34
Ciprofloxacin + Tinidazole	J01RA*73	-	-	-	19	-	-	-	-	-	-	-	-
Cefoperazone + Sulbactam	J01RA*83	-	259	-	30	-	-	-	56	-	16	-	159
Ceftriaxone + Sulbactam	J01RA*84	-	315	-	159	-	-	-	177	-	63	-	253
Ceftriaxone + Tazobactam	J01RA*85	-	537	-	352	-	2000	-	916	-	242	-	392
Cefotaxime + Sulbactam	J01RA*86	-	-	-	193	-	-	-	278	-	120	-	41
Cefixime + Ofloxacin	J01RA*91	-	833	-	-	-	-	-	-	-	127	-	-
Ofloxacin + Metronidazole	J01RA*72	-	-	-	-	-	2456	-	82	-	-	-	87
Metronidazole (parenteral)	J01XD01	1960	259	1088	104	-	-	2643	131	911	52	2066	152
Metronidazole (oral)	P01AB01	2105	-	1381	-	2571	-	1560	-	331	-	1166	-

* Sulfamethoxazole + trimethoprim, Abbreviations ATC = The Anatomical Therapeutic Chemical Classification System; DDD = Defined Daily Dose; J01RA* = ATC codes according to Sharma et al. [17]; NTH = Nonteaching hospital; OBGY = Obstetrics and Gynaecology departments; TH = Teaching hospital.
